# Supplementary material for: Burden of scabies in a Ghanaian penitentiary
Source: PLoS One. 2024 Dec 6;19(12):e0312108. doi: 10.1371/journal.pone.0312108 (PMC11623463; doi:10.1371/journal.pone.0312108)
Supplement: Attachment — Submitted filename: Response to Reviewers_R2.docx [file pone.0312108.s001.docx]

8^th^ September 2024

**Kumasi Centre for Collaborative Research**

**Kwame Nkrumah University of Science and Technology**

**Kumasi, Ghana**

**The Editor**

**PLOS One Journal**

Dear Sir,

**Re: ‘Burden of scabies in a Ghanaian penitentiary’ [PONE-D-24-01158R1] - [EMID:dc590fc36ba29ccb]**

My co-authors and I have taken note of the review comments and revised the manuscript as suggested.

We wish to submit the revised manuscript for publication in your esteemed journal. In the attached reply, the reviewer questions are in blue font and our responses are in red font.

We look forward to the next steps towards the publication of our manuscript.

Yours Sincerely,

Dr Yaw Ampem Amoako

Corresponding author

**Point-by-point response to review comments**

Journal Requirements:

Please review your reference list to ensure that it is complete and correct. If you have cited papers that have been retracted, please include the rationale for doing so in the manuscript text, or remove these references and replace them with relevant current references. Any changes to the reference list should be mentioned in the rebuttal letter that accompanies your revised manuscript. If you need to cite a retracted article, indicate the article’s retracted status in the References list and also include a citation and full reference for the retraction notice.

Response: Done

Additional Editor Comments:

The two reviewers have different views on the current status of your manuscript. Reviewer 2 holds concerns that their queries were not adequately addressed in the first round of revisions and that this prevents the work meeting PLOS ONE publication criteria. Please review the round 1 comments. along with any here, and clearly and fully address these points.

Response: We thank the Editor and the reviewers for making time to review our manuscript. We have taken note of the comments and have worked to address the concerns. The manuscript has been revised according.

Comments to the Author

1. If the authors have adequately addressed your comments raised in a previous round of review and you feel that this manuscript is now acceptable for publication, you may indicate that here to bypass the “Comments to the Author” section, enter your conflict of interest statement in the “Confidential to Editor” section, and submit your "Accept" recommendation.

Reviewer #1: All comments have been addressed

Reviewer #2: (No Response)

Response: Thank you.

2. Is the manuscript technically sound, and do the data support the conclusions?

The manuscript must describe a technically sound piece of scientific research with data that supports the conclusions. Experiments must have been conducted rigorously, with appropriate controls, replication, and sample sizes. The conclusions must be drawn appropriately based on the data presented.

Reviewer #1: Yes

Reviewer #2: Partly

Response: Thank you.

6. Review Comments to the Author

Please use the space provided to explain your answers to the questions above. You may also include additional comments for the author, including concerns about dual publication, research ethics, or publication ethics. (Please upload your review as an attachment if it exceeds 20,000 characters)

Reviewer #1: Congratulations to the authors for coming up with this revision. All the comments dealt with appropriately

Response: Thank you.

Reviewer #2: Despite the revisions, significant flaws are still exist. Many of the previously raised concerns and comments have not been adequately addressed in the revised manuscript. As the persistence of these issues and the authors' inability to address them adequately, I regret to inform you that the manuscript cannot be accepted for publication in its current form. I encourage the authors to carefully consider and address the comments in any future resubmissions.

Response: Thank you. We have taken note of the comments of the reviewer. We have revised the manuscript based on the comments from the first round of revisions as detailed below.

Reviewer #2: Major Comments:

1. Authors reported the scabies prevalence of 65.8% among inmates which is significantly higher. Authors are suggested not rely only on clinical diagnosis but also prefer confirmatory tests like dermoscopy, culturing, PCR etc. especially in such a high prevalence rate.

Response: We thank the reviewer for the comment.

We used the consensus criteria for the clinical diagnosis of scabies established by the International Alliance for Control of Scabies (IACS) for scabies diagnosis in the current study. This criteria developed via a Delphi study, showed high agreement among experts (*Engelman D, Fuller LC, Steer AC. Consensus criteria for the diagnosis of scabies: A Delphi study of international experts. PLoS Negl Trop Dis. 2018;12(5):e0006549*; *Engelman D, Yoshizumi J, Hay RJ, et al. The 2020 International Alliance for the Control of Scabies Consensus Criteria for the Diagnosis of Scabies. Br J Dermatol. 2020 Nov;183(5):808-820)* and has been validated and shown subsequently to be useful for scabies diagnosis in varied field settings (Walker SL, Collinson S, Timothy J, et al. A community-based validation of the International Alliance for the Control of Scabies Consensus Criteria by expert and non-expert examiners in Liberia*. PLoS Negl Trop Dis. 2020 Oct 5;14(10):e0008717;* Matthews A, Le B, Amaral S, et al. Prevalence of scabies and impetigo in school-age children in Timor-Leste. *Parasit Vectors. 2021 Mar 15;14(1):156;* Amoako YA, Phillips RO, Arthur J, et al. A scabies outbreak in the North East Region of Ghana: The necessity for prompt intervention. *PLoS Negl Trop Dis. 2020 Dec 22;14(12):e0008902*).

Furthermore, the IACS criteria is recommended for the diagnosis of scabies in implementation research and mapping projects, and for surveillance after control interventions (Engelman D, Fuller LC, Steer AC. Consensus criteria for the diagnosis of scabies: A Delphi study of international experts*. PLoS Negl Trop Dis. 2018;12(5):e0006549)*.

While dermoscopy allows for direct visualization of the scabies mites, a systematic review of diagnostic methods of scabies reported low accuracy of dermoscopy in diagnosing scabies and clinical diagnosis remains an accepted practice in field studies *(*Leung V, Miller M. Detection of scabies: A systematic review of diagnostic methods. *Can J Infect Dis Med Microbiol. 2011;22(4):143-6*; Engelman D, Yoshizumi J, Hay RJ, et al. The 2020 International Alliance for the Control of Scabies Consensus Criteria for the Diagnosis of Scabies. *Br J Dermatol. 2020 Nov;183(5):808-820*).

In addition, dermoscopy is not readily available in resource limited settings like ours where the study was conducted. Further, dermoscopy is more likely to be useful tool in settings with lower number of patients to screen and a lower pretest likelihood of scabies than in an outbreak setting.

Indeed, we are unaware of any recommendation regarding using PCR for scabies diagnosis in standard care; and culture may be performed for confirming superinfection when impetigo is present.

In low resource settings where scabies prevalence is highest, tools like PCR and others mentioned by the reviewer are not readily available and clinical diagnosis using criteria like those proposed by IACS have been shown in multiple studies to be adequate for implementation research and surveillance (Engelman D, Fuller LC, Steer AC. Consensus criteria for the diagnosis of scabies: A Delphi study of international experts*. PLoS Negl Trop Dis. 2018;12(5):e0006549;* Walker SL, Collinson S, Timothy J, et al. A community-based validation of the International Alliance for the Control of Scabies Consensus Criteria by expert and non-expert examiners in Liberia*. PLoS Negl Trop Dis. 2020 Oct 5;14(10):e0008717;* Matthews A, Le B, Amaral S, Arkell P, et al. Prevalence of scabies and impetigo in school-age children in Timor-Leste. *Parasit Vectors. 2021 Mar 15;14(1):156)*.

We have also included in the limitations section (line 354-357) the following sentences:

‘We did not perform molecular based tests like PCR nor use tools like cultures for laboratory confirmation of fungal or bacterial infections. No skin biopsies were taken for histological examination. These tests are expensive and not routinely available to the population in which the current study was conducted.’

2. Authors reported that a significant percentage (79.2%) of previously treated individuals still showed skin manifestations of scabies. This raises questions about treatment efficacy and possibility of reinfection and its potential factors. Authors are suggested to discuss the potential reasons behind this observation/treatment failure. It would be appropriate to include the exploring strategies for more effective treatment.

Response: We thank you for the comment. We included the requested information in the discussion section. We state that:

‘In our study, 17% of participants had received treatment for their skin problems in the 2 months preceding the assessment. However, 79.2% of the individuals who were previously treated still exhibited skin manifestations of scabies; these may include individuals with post scabies itch, reinfections/ recurrences or non-response to treatment/ treatment failure. There had been no treatment of the contacts of previously identified scabies cases. The lack of a systematic approach to the treatment of previously identified scabies cases and their contacts likely led to reinfection of individuals and ongoing spread within the prison.’ (lines 275-281).

We have also added the following sentences to the discussion section (line 281-285).: ‘Additionally, the overcrowded living conditions, limited access to health care and inadequate Water, Sanitation and Hygiene (WASH) facilities may have facilitated scabies spread within the prison. There is the need to explore strategies for more effective control of scabies within this prison setting.’

Regarding strategies for effective scabies control, we included the sentences (lines 290-317):

‘There are different strategies for scabies control. A control strategy of treating clinically affected persons and their contacts may provide temporary relief for the individuals but has limited success in reducing population prevalence in the longer term [27].

Mass drug administration (MDA) using topical permethrin or oral ivermectin offers an alternative approach for population control to substantially reduce the burden of scabies.

Mass treatment of highly endemic communities with topical 5% permethrin has been reported to substantially reduce scabies prevalence [28-31]. In another study with permethrin, although the scabies prevalence remained unchanged, the prevalence of secondary infected scabies decreased from 3.7% to 1.5% indicating a relative reduction of 59% [32].

Topical permethrin is expensive and requires application to the whole body. Other disadvantages of topical agents include inadequate application to local lesions, a lack of privacy to undertake whole-body application, insufficient facilities to rinse off the cream, poor compliance among contacts of scabies cases and discomfort due to topical treatment [33]. These factors constitute major barriers for effective use of topical therapy for population control of scabies, especially in the prison setting.

Ivermectin, as an oral therapy can offset some of the challenges associated with topical treatment and can potentially increase compliance resulting in better control of scabies in affected populations [8, 34]. Studies on MDA with ivermectin performed in island settings [15, 16, 35] reported a reduced prevalence of scabies. Compared to topical permethrin, oral ivermectin resulted in larger reductions in scabies prevalence at 12 and 24 months [15]. Recently, mass screening and community treatment (MSAT) of cases and their contacts with oral ivermectin delivered by community health workers has been shown to be superior to mass screening followed by usual care involving referral to clinic for topical treatment in controlling scabies in India [34]. While MSAT with ivermectin showed promising results and has relatively lower costs, the approach might miss persons with subclinical scabies making it less effective than MDA [34]. Furthermore, MSAT does not target people who work in the prison and may be contacts of scabies cases.’

3. Authors stated the (MDA with ivermectin) as a potential solution, authors are suggested to include the potential drawbacks of this approach. Authors may add alternative combination approaches for scabies control.

Response: Thank you for this comment. The section on approaches to population control of scabies reads (lines 290-325):

‘There are different strategies for scabies control. A control strategy of treating clinically affected persons and their contacts may provide temporary relief for the individuals but has limited success in reducing population prevalence in the longer term [27].

Mass drug administration (MDA) using topical permethrin or oral ivermectin offers an alternative approach for population control to substantially reduce the burden of scabies. Mass treatment of highly endemic communities with topical 5% permethrin has been reported to substantially reduce scabies prevalence [28-31]. In another study with permethrin, although the scabies prevalence remained unchanged, the prevalence of secondary infected scabies decreased from 3.7% to 1.5% indicating a relative reduction of 59% [32].

Topical permethrin is expensive and requires application to the whole body. Other disadvantages of topical agents include inadequate application to local lesions, a lack of privacy to undertake whole-body application, insufficient facilities to rinse off the cream, poor compliance among contacts of scabies cases and discomfort due to topical treatment [33]. These factors constitute major barriers for effective use of topical therapy for population control of scabies, especially in the prison setting.

Ivermectin, as an oral therapy can offset some of the challenges associated with topical treatment and can potentially increase compliance resulting in better control of scabies in affected populations [8, 34]. Studies on MDA with ivermectin performed in island settings [15, 16, 35] reported a reduced prevalence of scabies. Compared to topical permethrin, oral ivermectin resulted in larger reductions in scabies prevalence at 12 and 24 months [15]. Recently, mass screening and community treatment (MSAT) of cases and their contacts with oral ivermectin delivered by community health workers has been shown to be superior to mass screening followed by usual care involving referral to clinic for topical treatment in controlling scabies in India [34]. While MSAT with ivermectin showed promising results and has relatively lower costs, the approach might miss persons with subclinical scabies making it less effective than MDA [34]. Furthermore, MSAT does not target people who work in the prison and may be contacts of scabies cases.

We found a scabies prevalence in the prison which is much higher than the 10% threshold set for MDA [36]. In the current study, individuals with scabies were treated with topical benzyl benzoate as recommended in Ghana. However, the high prevalence and large number of contacts (entire prison population including staff and health workers) make MDA with ivermectin imperative. An urgent collaborative effort involving multiple stakeholders such as the Ghana Prisons Service, the Ghana Health Service, civil society and non-governmental organisations and donors is needed to mobilise adequate doses of ivermectin to undertake an MDA in this institutionalized setting.’

We have revised the section on the drawbacks of ivermectin MDA in the discussion and it now reads as follows (line 326-340):

‘Ivermectin is contraindicated in children < 5 years old, persons with severe concurrent illness, individuals with hypersensitivity to components of the drug, and pregnancy. Inadequate community engagement, lack of political support and high costs are potential barriers that may pose challenges for MDA implementation [37, 38]. Furthermore, due to its lack of ovicidal action, a second dose of ivermectin is usually recommended after 7-14 days for individual treatment, to kill newly hatched mites. Administration of a second dose that is separated by several days as MDA is logistically challenging and is associated with higher costs. An oral agent with a longer duration of activity in the skin, that could persist for sufficient time to kill newly hatched mites, would make the requirement for a second dose unnecessary. Moxidectin with a half-life of up to 43 days, and prolonged activity in the skin offers promise in this regard [39]. An initial preclinical study reported that oral single dose moxidectin was more effective than two consecutive doses of ivermectin for treating scabies [40]. In future moxidectin may be even more helpful as its longer half-life may render it more efficient in the prison setting where interactions between people are not restricted by geographic boundaries as exists in island populations.’

4. There are already similar studies have been published. Therefore, authors should justify the objectives and novelty of the current study.

Response: As alluded to by reviewer 1, ‘’As yet, there seems not to be any data on this topical issue in Ghanaian prisons.’’

This study provides important data on scabies within the Ghanaian prison context. It is the first study that has utilised the IACS criteria for diagnosing scabies in the prison context. (line 104-108)

5. What were the inclusion and exclusion criteria? It would be more appropriate to add sample size calculation and its justification? As sample size is vital to represent the study population.

Response: Thank you for this comment. We have indicated in the methods section (line 141-145) that:

‘A convenience sampling technique was employed to identify and select participants for the study. We aimed to include one third of all inmates in the study. To achieve this, inmates from each cell or dormitory within the prison were made to stand in line and every third prisoner from who provided informed consent was enrolled.’

We further added the following sentence to the section (line 145-146):

‘Individuals who did not provide informed consent were excluded from the study.’

6. Explain if the questionnaire was pilot tested. Have the participants completed an informed consent? It remains to include the study population, the sample and the sampling.

Response: We agree informed consent to be extremely important in the prison setting. The consent procedure is described in the methods as part of the ethical considerations (line 110-123). Written informed consent was obtained from all participants.

The study setting, participant recruitment and sampling procedures were described in the methods section (line 124-181).

We have clarified that a convenience sampling approach was used to select participants for this study. (line 141-146)

We have further added the following sentences to the ethics section:

‘Participant autonomy, confidentiality, and welfare were always maintained and given the highest priority. At any time, study participants had the option of requesting withdrawal and removal of their coded data from the study without it affecting their treatment. Both inmates participating in the study diagnosed with scabies and inmates not participating in study reporting to study team with scabies symptoms were treated. All study procedures conformed with the principles guiding research in human subjects as set out in the Declaration of Helsinki [21]. (line 117-123)

7. Authors should describe limitations of their study. Furthermore, these limitations should be discussed.

Response: Thank you for the comment. We have revised the section on ‘study strengths and limitations’ and it now reads (line 342-357):

‘Our study included a relatively large number of individuals and is the first systematic assessment of the burden of scabies among prisoners in Ghana. The study was conducted in the second largest prison in the country and similar outbreaks may be ongoing in other prisons in Ghana. We used the IACS criteria for standardisation of scabies diagnosis and this was easily and effectively applied in this resource limited setting in Ghana. However, there are also certain limitations. The diagnosis of scabies burrows was not confirmed with dermoscopy. However, a systematic review of diagnostic methods of scabies has shown low accuracy of dermoscopy in diagnosing scabies and clinical diagnosis remains an accepted practice in field studies [41]. In addition, dermoscopy is not readily available in most resource limited settings. Furthermore, the IACS criteria have not been validated in the prison setting where there may be a different clinical background of skin problems than the settings the IACS criteria was validated in. We did not perform molecular based tests like PCR nor use tools like cultures for laboratory confirmation of fungal or bacterial infections. No skin biopsies were taken for histological examination. These tests are expensive and not routinely available to the population in which the current study was conducted.’

Additional Comments:

1. Proof read the article to improve sentence structure.

Response: Done as suggested.

2. Read similar articles to polish your write-up. e.g. https://www.mdpi.com/1648-9144/59/11/1905. This article may help to improve your understandings and quality of article.

Response: We are grateful to the reviewer for the reference. We have included this in the list of references (#14).
